# Molecular Pathology and Targeted Therapies for Personalized Management of Central Nervous System Germinoma

**DOI:** 10.3390/jpm11070661

**Published:** 2021-07-14

**Authors:** Cristina Ilcus, Horatiu Silaghi, Carmen Emanuela Georgescu, Carmen Georgiu, Anca Ileana Ciurea, Simona Delia Nicoara, Cristina Alina Silaghi

**Affiliations:** 1 Department of Endocrinology, County Clinical Emergency Hospital Cluj, 3–5 Clinicilor Street, 400006 Cluj-Napoca, Romania; cristina.ilcus@yahoo.com; 2 Department of Surgery V, “Iuliu Hațieganu” University of Medicine and Pharmacy Cluj-Napoca, 8 Victor Babes Street, 400012 Cluj-Napoca, Romania; 3 Department of Endocrinology, “Iuliu Hațieganu” University of Medicine and Pharmacy Cluj-Napoca, 8 Victor Babes Street, 400012 Cluj-Napoca, Romania; c_e_georgescu@yahoo.com (C.E.G.); alinasilaghi@yahoo.com (C.A.S.); 4 Department of Pathological Anatomy, “Iuliu Hațieganu” University of Medicine and Pharmacy Cluj-Napoca, 8 Victor Babes Street, 400012 Cluj-Napoca, Romania; carmengeorgiu@hotmail.com; 5 Department of Radiology, “Iuliu Hațieganu” University of Medicine and Pharmacy Cluj-Napoca, 8 Victor Babes Street, 400012 Cluj-Napoca, Romania; ancaciurea@hotmail.com; 6 Department of Ophthalmology, “Iuliu Hațieganu” University of Medicine and Pharmacy, 8 Victor Babes Street, 400012 Cluj-Napoca, Romania; simonanicoara1@gmail.com

**Keywords:** CNS germinoma, KIT, RAS, MAPK, miRNAs, radiotherapy, chemotherapy, personalized targeted therapy

## Abstract

Intracranial germinomas are rare tumours, usually affecting male paediatric patients. They frequently develop in the pineal and suprasellar regions, causing endocrinological disturbances, visual deficits, and increased intracranial pressure. The diagnosis is established on magnetic resonance imaging (MRI), serum and cerebrospinal fluid (CSF) markers, and tumour stereotactic biopsy. Imaging techniques, such as susceptibility-weighted imaging (SWI), T2* (T2-star) gradient echo (GRE) or arterial spin labelling based perfusion-weighted MRI (ASL-PWI) facilitate the diagnosis. Germinomas are highly radiosensitive tumours, with survival rates >90% in the context of chemoradiotherapy. However, patients with resistant disease have limited therapeutic options and poor survival. The aim of this review is to highlight the genetic, epigenetic, and immunologic features, which could provide the basis for targeted therapy. Intracranial germinomas present genetic and epigenetic alterations (chromosomal aberrations, *KIT*, *MAPK* and *PI3K* pathways mutations, DNA hypomethylation, miRNA dysregulation) that may represent targets for therapy. Tyrosine kinase and *mTOR* inhibitors warrant further investigation in these cases. Immune markers, PD-1 (programmed cell death protein 1) and PD-L1 (programmed death-ligand 1), are expressed in germinomas, representing potential targets for immune checkpoint inhibitors. Resistant cases should benefit from a personalized management: genetic and immunological testing and enrolment in trials evaluating targeted therapies in intracranial germinomas.

## 1. Introduction

Intracranial germ cell tumours (ICGCT) are rare tumours that primarily affect children and adolescents, with a male predominance, accounting for 3.6% of brain tumours in Western Europe and reaching a higher incidence of 15.4% in Japan [1,2,3]. A comparative study between Japanese and American populations regarding ICGCT revealed a different distribution of the tumours. More cases of basal ganglia involvement were present in the Japanese, whereas more bifocal (synchronous pineal and suprasellar) locations in western society, suggesting the presence of genetic or environmental factors, thus, contributing to the phenotypic diversity [4]. According to the WHO classification of the central nervous system (CNS) tumours, the germ cell tumours group, composed of germinoma, represents the most common histological type (60%), followed by mixed germ cell tumours (17.4%), teratoma (15.7%), yolk sac tumour (4.2%), choriocarcinoma (1.6%), and embryonal carcinoma (1.1%) [5]. According to Matsutani et al., pure germinoma and mature teratoma have a good prognosis, but other histological types may result in unfavourable prognosis. Intermediate prognosis corresponds to germinoma with syncytiotrophoblastic giant cell [STGC], immature teratoma, teratoma with malignant transformation and mixed tumours composed mainly of germinoma or teratoma, whereas poor prognosis is found in choriocarcinoma, yolk sac tumour, embryonal carcinoma, as well as in mixed tumours composed mainly of choriocarcinoma, yolk sac tumour, or embryonal carcinoma [6,7]. 

Germinomas usually develop in the midline areas of the brain, most often in the pineal gland (50% of the pineal tumours are germinomas) and the suprasellar region [8]. In approximately 5–10% of the cases, the tumour is ectopically situated (in other areas than neurohypophysial or pineal sites), including the basal ganglia, thalamus, corpus callosum, cerebellum, septum pellucidum, temporal lobe, and the spinal cord [9,10,11,12,13]. Suprasellar germinomas are associated with female patients (<15 years old), while pineal germinomas with male patients (>15 years) [14]. In rare cases, germinomas have been described to occur later in life. Several reports mention the diagnosis in the sixth or seventh decade of life, outlining the fact that germinoma should be included as a differential diagnosis even in the elderly [15,16,17,18,19]. Germinomas can also be present as synchronous lesions in the pineal region and hypothalamo-neurohypophyseal axis, also referred to as “bifocal germinomas”, with an incidence ranging from to 6% to 26% and even as high as 41% in some studies [20,21,22,23,24]. Metastatic germinomas caused by the dissemination of tumour cells through the cerebrospinal fluid are reported in approximately 4.5% of the cases, usually after a mean period of 6–7 years after the initial tumour diagnosis [25,26]. According to the literature, the most common type of metastasis is spinal “drop metastasis” (32.5%), followed by ventricular dissemination (30%), and to a lesser extent, the suprasellar region, corpus callosum, subarachnoid space [26,27,28]. Concomitant metastases to the ventriculoperitoneal shunt can appear in 20% of the cases [26].

## 2. Clinical Presentation

Tumours of the pineal gland obstruct the posterior wall of the third ventricle and of the aqueduct of Sylvius, resulting in acute hydrocephalus with headaches, nausea, projectile vomiting, papilledema, and lethargy. Generally, a 2 cm pineal tumour can cause obstructive hydrocephalus [29]. When the tumour grows, it has a compressive effect on the nerve pathway connecting the cortex to the oculomotor nuclei and the superior colliculi, resulting in Parinaud’s syndrome: upward gaze palsy, pupillary reflex dysfunction, and convergence-retraction nystagmus [30]. In comparison with other types of pineal tumours, upward gaze palsy seems to be more frequently encountered in pure germinomas (90%), as a consequence to the mesencephalic dysfunction caused by the germinoma’s progression pattern [31]. In cases of pituitary germinoma, visual disturbances can result from optic chiasma compression or perioptic meningeal seeding [32]. 

Germinomas developed in the suprasellar region or anterior third ventricle may be presented with endocrinopathy at the diagnosis (diabetes insipidus, delayed growth or gonadal function, precocious puberty, menstrual irregularities, visual field/acuity deficits). Neurohypophyseal axis dysfunction may also appear as an adverse effect of the radiotherapy treatment [21,30,33,34,35]. Diabetes insipidus (DI) is the most common symptom associated with germinomas occurring in the hypothalamic-neurohypophysial region, followed by visual deficits, hypopituitarism, and increased intracranial pressure [36]. Diabetes insipidus seems to be a reliable predictor of tumoral invasion of the hypothalamus and third ventricle even in the absence of MRI evidence of suprasellar and third ventricle disease [37,38,39]. GH (growth hormone) deficiency and hypogonadism are the most frequent endocrine insufficiencies (89–95% of cases), followed by hypothyroidism and hypocortisolaemia, in approximately 50% of patients [40]. Boys with intracerebral germ tumours can develop precocious puberty (isosexual pseudoprecocity) because of beta-human chorionic gonadotropin (β-hCG) secretion of the syncytiotrophoblasts component of the tumour, which stimulates Leydig cells, with subsequent production of testosterone [41,42].

Patients with basal ganglia/thalamus germinoma often develop hemiparesis, headache, ataxia, cognitive impairment, and mental status alterations, though the severity of clinical picture does not seem to correlate with the tumour size [43,44]. Cases of optic nerve germinoma, although rare, present with progressive visual deficits, followed by endocrine dysfunction (most often DI), symptoms that may be inconsistent with the MRI findings, causing a delay in diagnosis [45].

A retrospective study on 49 children diagnosed with pure germinomas reported visual impairments as the most common symptom (47.9%), followed by motor dysfunctions (40.8%), frequently including focal or general weakness, hemiparesis, facial palsy. Patients presenting endocrinological symptoms had a significant delay in diagnosis [39]. Approximately one-third of the patients have prolonged symptomatology (>6 months) before the diagnosis, these cases being associated with a higher risk of metastatic disease [46]. A summary of the clinical presentation is shown in Table 1.

## 3. Diagnosis

### 3.1. Biological Markers

Different biomarkers have been studied in order to establish the optimum diagnosis. A relative correlation between serum and cerebrospinal fluid (CSF) biomarkers and the tumour’s histological category was set. Alpha-fetoprotein (AFP) is elevated in embryonal carcinomas and teratomas, whereas choriocarcinomas and germinomas secrete β-hCG [47,48]. However, germinomas have an inconsistent secretion of β-hCG [49,50]. There is no clear cut-off for hCG levels to distinguish germinomas from mixed germ tumours, but it is considered that pure germinomas produce no or mild levels of CSF β-hCG (<50 mUI/mL), in the latter cases being classified as high risk and requiring a more aggressive chemotherapy regimen [37,51]. A study conducted on 80 germ cell tumours revealed a sensitivity of 78.9% and a specificity of 96.6% for CSF tumour markers (with a cut-off of 50 IU/L for ß-HCG and 25 ng/mL for AFP). Marker positive germinomas, as well as marker negative NGGCT have been reported [52].

**Figure 1:**
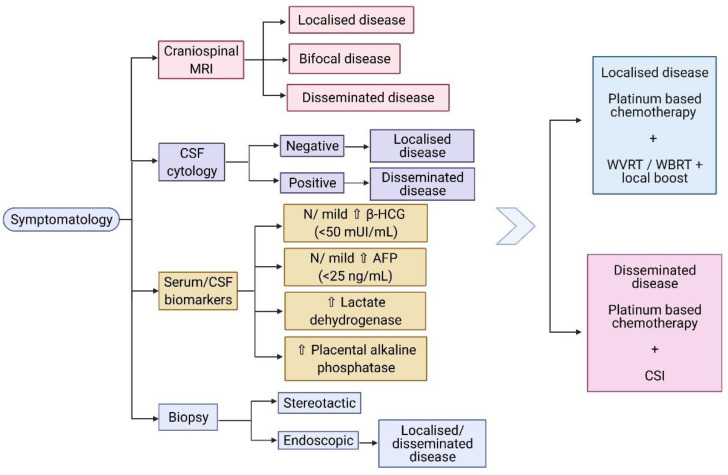
(Created with BioRender.com) Diagnostic algorithm of CNS germinomas. Abbreviations: MRI: magnetic resonance imaging; CSF: cerebrospinal fluid; β-HCG: beta- human chorionic gonadotropin; AFP: alpha fetoprotein; N: normal; WVRT: whole ventricular radiotherapy; WBRT: whole brain radiotherapy; CSI: craniospinal irradiation.

Other biomarkers associated with germinomas include elevated lactate dehydrogenase (LDH) and placental alkaline phosphatase (PLAP). These biomarkers, in combination with the radiological finding of a pineal mass, could provide a high suspicion of the histological subtype, especially in cases of heterogeneous tumours, and may represent a way of monitoring the treatment response [53]. Aihara et al. discovered that the CSF PLAP level is a specific marker for pure germinomas, which can provide a reliable diagnosis of intracranial germinoma, in the absence of a histopathological examination [54]. However, Chiba et al. propose that CSF PLAP levels, with a cut-off value of 8 pg/mL, also correlate with the germinoma component in the context of mixed GCT [55]. The diagnostic algorithm of CNS germinomas, including the main serum/CSF biomarkers, is shown in Figure 1.

### 3.2. Radiological Characteristics

**Figure 2:**
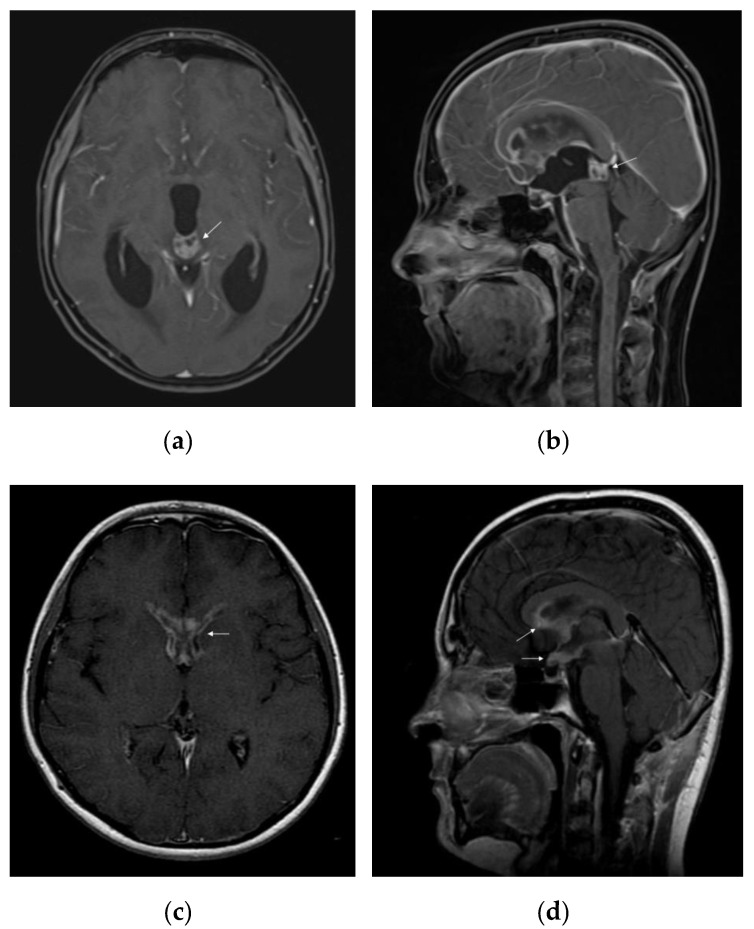
MRI features of intracranial germinoma in a teenage patient: (**a**) pineal germinoma: heterogeneous contrast enhancement on axial gadolinium-enhanced T1-weighted image, with a tendency to cardioid shape; (**b**) pineal germinoma: heterogeneous contrast enhancement on sagittal gadolinium-enhanced T1-weighted image; (**c**) postoperative disseminated disease: bilateral nodular enhancement of anterior horns of the lateral ventricles on axial gadolinium-enhanced T1-weighted image; (**d**) postoperative disseminated disease: nodular enhancement of the hypothalamus, optic chiasm, mammillary bodies, and corpus callosum on sagittal gadolinium-enhanced T1-weighted image.

On standard MRI, germinomas appear as heterogeneous tumours in T1/T2-weighted imaging, in 40% of the cases, and the uptake of the gadolinium can be either homogeneous (47%) or heterogeneous (53%) [56]. Relevant images of a pure pineal germinoma are shown in Figure 2. 

Multiple imaging studies have been conducted to provide a better characterization of germinomas. For example, Inoue et al. showed that 90% of the patients with pineal germinomas presented a cardioid-shape tumour image on the axial MRI views, due to its progression pattern on both sides of the third ventricle, concluding that this was a specific aspect for pure pineal germinoma [31]. Awa et al. described two significant features differentiating pineal germinomas from NGGCT: peritumoural edema thicker than 5 mm (peritumoural area with T2 hyperintensity) and bithalamic extension [57]. T2* (T2-star) sequence is generally used to obtain a better characterization of intratumoural/intraventricular/cerebral microhaemorrhage, iron deposits, and calcifications [58]. Susceptibility-weighted imaging (SWI) or T2* gradient echo (GRE) technique can be used for better differentiation between pure germinoma and NGGCT in the pineal region: 93% of the germinomas present iso- or hyperintensity, whereas NGGCT are hypointense compared to the healthy brain [56]. Another imaging technique, such as the arterial spin labelling based perfusion-weighted MRI (ASL-PWI) could be used in differentiating germinomas from NGGCT, based on lower values of relative tumour blood flow encountered in germinomas [59]. Calcification can be present in both germinomatous and non-germinomatous pineal tumours [56]. 

Suprasellar germinomas seem to develop from the tuber cinereum and median eminence, infiltrating the infundibulum [60]. Therefore, an isolated thickened pituitary stalk may be the first radiological appearance of a hypothalamo-hypophyseal germinoma [61,62]. However, they have a delay in diagnosis of a median of 1.4 years, due to insidious onset of symptomatology and MRI findings, often suggestive of inflammation (lymphocytic hypophysitis), pituitary adenomas, and secondary neoplasms, with radiological appearance similar to germinomas [63,64,65]. Nonetheless, GCT represent 66.7% of widened pituitary stalk causes in paediatric population, whereas germinomas represent the second etiology (21–31%) of enlarged pituitary stalk in adults [62,66]. Cases of diabetes insipidus followed by the occurrence of germinoma during the MRI follow-up have been described, highlighting the importance of imaging re-examination or endoscopic biopsy (with higher sensitivity compared to imaging studies) [38,61]. Usually, pituitary stalk infiltration is reversible following adequate treatment [60]. 

Basal ganglia and thalamus germinomas may present variable neuroimaging features (cystic lesion, peritumoural oedema, calcification, intratumoural haemorrhage, contrast enhancement, ipsilateral cerebral atrophy) that may impede reaching the correct diagnosis. Nonetheless, ipsilateral hemiatrophy seems to be a characteristic feature of basal ganglia and thalamus germinomas, which may differentiate them from other tumour types [44]. Surprisingly, the number of lesions detected on the MRI does not represent a poor prognosis factor and does not correlate with the overall survival in the setting of an appropriate treatment protocol [67].

### 3.3. Biopsy

In the management of germinomas, most studies recommend stereotactic biopsy for a definite diagnosis. Balossier et al. have shown that the histopathological diagnosis for pineal biopsies is more accurate with stereotactic procedures than with endoscopic procedures (93.7% vs. 81.1%) [68]. However, several studies emphasized the importance of endoscopic diagnosis, since in patients with pineal germinoma and DI, metastatic lesions to the third ventricular floor are more frequently identified by direct endoscopy than initially diagnosed by MRI [37,38]. Accordingly, biopsy-diagnosed pineal germinoma in DI patients should be classified as disseminated disease, even in the absence of MRI evidence [38]. Still, a retrospective multicentre study evaluated the necessity of performing biopsy in patients with bifocal tumour, diabetes insipidus, and negative tumour markers. The study included 91 patients with available histologic diagnosis, of which 92% were pure germinomas and germinomas with syncytiotrophoblastic giant cells, concluding that a tumour biopsy is recommended to ensure the proper diagnosis [69]. Another retrospective study revealed that in cases of pituitary germinoma suspected on MRI, the biopsy can reveal another pathology in 22% of the tumours [70]. Moreover, a fairly recent technique that combines endoscopic biopsy with endoscopic ventriculostomy, using a single trajectory, is considered safe and could become an alternative for the dual procedure in pineal germinomas [71]. 

### 3.4. Histological Diagnosis

Macroscopically, germinomas are solid, soft, grey-white, homogenous tumours; however, they can rarely present areas of haemorrhage, necrosis, or cystic components. They can be variably encapsulated or poorly circumscribed and infiltrative. Microscopically, they consist of large primordial germ cells (undifferentiated cells), with clear, abundant PAS+ cytoplasm, large, round nuclei, and prominent nucleoli; occasionally syncytiotrophoblastic giant cells may be present [72]. The cells have high mitotic activity and are organized in sheets, lobules, or nests patterns, separated by fibrovascular septae filled with lymphocytic infiltrates. Occasionally, the lymphoplasmacellular reaction is so robust that the granulomatous inflammation can obscure the tumour cells [5]. Therefore, a characteristic histopathological feature of germinoma is the “two-cell pattern”: a massive immune cell population, with a high lymphocytic predominance, dispersed between tumour cells [73].

**Figure 3:**
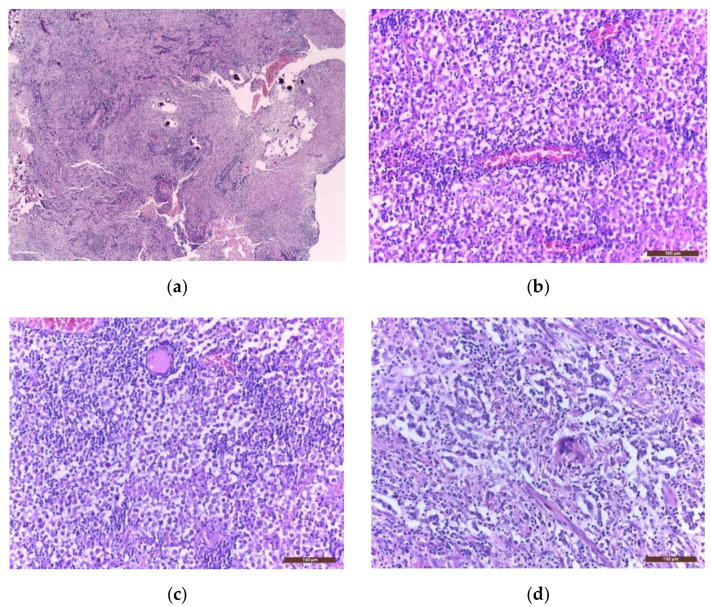
Microscopic images of extragonadal germinomas are identical with those of their gonadal counterpart: (**a**) pineal gland, with multiple small basophil psammoma bodies, infiltrated by a germinoma; (**b**) tumour proliferation with fine vascular network; (**c**) sheets of large germinoma cells, with pale cytoplasm, well defined cell membranes, large round central nuclei, and fibrous septae heavily infiltrated by lymphocytes; (**d**) an isolated multinucleated syncytiotrophoblast close to the centre of the image; (hematoxylin eosin staining; (**a**) 40× and (**b**–**d**) 200×).

**Figure 4:**
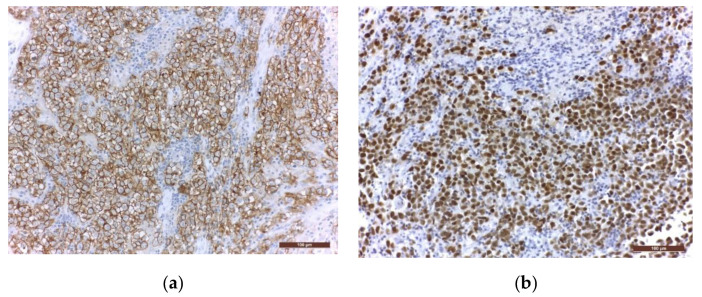

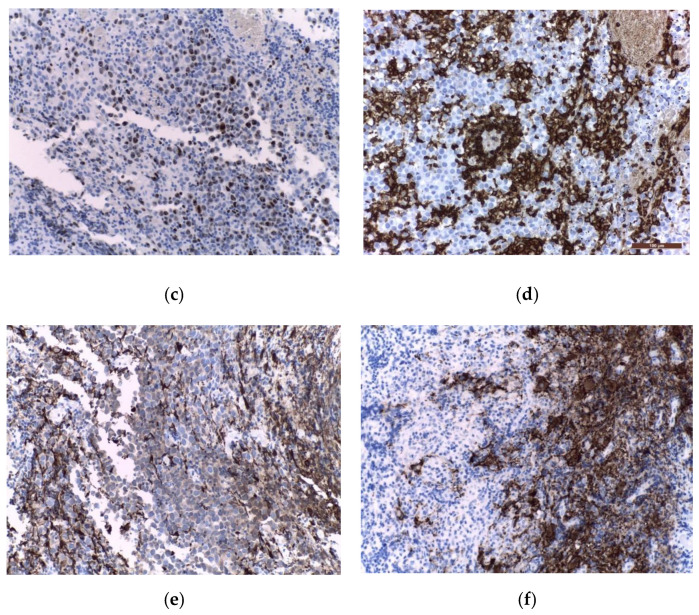
Immunohistochemical staining in extragonadal germinomas: (**a**) intense membranous and less intense cytoplasmic c-Kit/CD117 expression; (**b**) intense nuclear and less intense cytoplasmic OCT3/4 expression; (**c**) high Ki67 nuclear expression; (**d**) intense expression of leukocyte common antigen LCA/CD45 in stromal lymphocytes; (**e**) GFAP expression in glial pineal stromal cells in a case of pineal germinoma; (**f**) synaptophysin expression in the pinealocytes of a pineal germinoma; (200×).

Immunohistochemistry (IHC) is further used to provide the histological diagnosis. The membrane immunoreactivity for C-kit (transmembrane protein with tyrosine kinase activity), CD30 (tumour necrosis factor receptor), and D2-40 (podoplanin) aid in differentiating germinomas from embryonal carcinoma and yolk sac tumours [5]. The nucleus is usually reactive for OCT 3/4 (octamer binding transcription factor 3/4), SALL4 (sal-like protein 4), UTF1 (undifferentiated embryonic cell transcription factor 1), NANOG (transcription factor in embryonic stem cells), and ESRG (embryonic stem cell-related gene protein), whereas ribosomes are positive for LIN28 (RNA-binding protein LIN28) [74,75]. Although PLAP is a distinctive marker of primordial cells, its expression is less consistent, being detected in 82% of germinomas. On the other hand, C-kit and OCT 3/4 are more sensible, with 100% staining among germinoma cells. When the germinoma also contains a syncytiotrophoblastic component, these cells are positive for hCG, human placental lactogen (HPL), CD 30, and CK AE1/3 (cytokeratin AE1/3) [72]. A summary of IHC staining and representative histological images from intracranial germinomas are shown in Table 2 and Figure 3 and Figure 4. 

## 4. Staging

Though there are no standard staging criteria for germinomas, a modified Chang staging system is usually used, based on aspect imaging and serum/CSF markers. Localized disease on MRI (no evidence of metastasis) and negative CSF cytology are classified as M0, while intracranial/spinal metastasis or positive cytology classify the tumour as disseminated disease M+. Bifocal disease is defined by synchronous pineal and pituitary tumours [76]. Accordingly, an M1 stage presumes positive CSF cytology for tumour cells, and an M2 metastatic germinoma is defined by intracranial nodular seeding (except bifocal disease). The presence of spinal metastases includes the patient in the M3 stage and metastases outside CNS in the M4 stage [77]. 

## 5. Genetic Approach 

Germinomas have immunohistochemical and molecular alterations similar to testicular seminoma, suggesting a common pathogenesis. One theory regarding the origin of germinomas postulates that these tumours develop from primordial germ cells (PGCs) that mismigrated in the midline structures during early embryogenesis. This hypothesis is sustained by the global hypomethylation of germinoma cells, an epigenetic hallmark of PGCs, and by the presence of specific germ cells markers (c-Kit, Oct-3/4, and Nanog). Another theory proposes that germinomas arise from a pluripotent braincell, via *KIT* gene mutations [73]. There is little molecular data regarding germinoma’s development, considering the low incidence of this type of a tumour. Although rare, several familial cases of intracranial germinomas have been described, prompting further genetic studies regarding their tumourigenesis mechanism [78,79,80,81]. Fukushima et al. proposed that *MAPK* and/or *PI3K* pathway alterations, DNA hypomethylation, and chromosomal abnormalities represent a triad involved in the pathogenesis of pure germinomas [82].

A characteristic feature of germinomas that differentiates them from other ICGCT involves the methylation profile, germinomas presenting DNA hypomethylation, an acknowledged cause of genomic instability [82]. DNA methylation is a process normally involved in the epigenetic reprogramming of the germline during development, and, therefore, aberrant methylation patterns may have a significant role in the aetiology of germ cell tumours [83]. In a cohort of 54 germinomas, hypomethylation and KIT staining by immunohistochemistry were detected in 100% of the cases, underlining the resemblance to primordial germ cells [14]. Moreover, patients with chromosomal abnormalities (Down’s and Klinefelter syndrome) have been reported to develop CNS germinomas [84,85]. Chromosomal gains and losses are frequently encountered in germinomas (as presented in Table 3) [14,80,86,87]. Chromosomal aberrations such as gain of 2q and 8q and loss of 5q, 9p/q, 13q, and 15q are associated with a worse prognosis [7]. Wang et al. hypothesize that meiosis errors are involved in the pathogenesis of germinomas since 90% of the cases present chromosomal instability [87]. 

The most frequent genes involved in the pathogenesis of these tumours are the *KIT* and *RAS* genes, encountered in up to 40% and respectively 34.6% of germinomas (Table 4). Interestingly, *KIT* and *RAS* mutations are described as mutually exclusive in 97–100% of cases [14,86,87,88]. 

**Figure 5:**
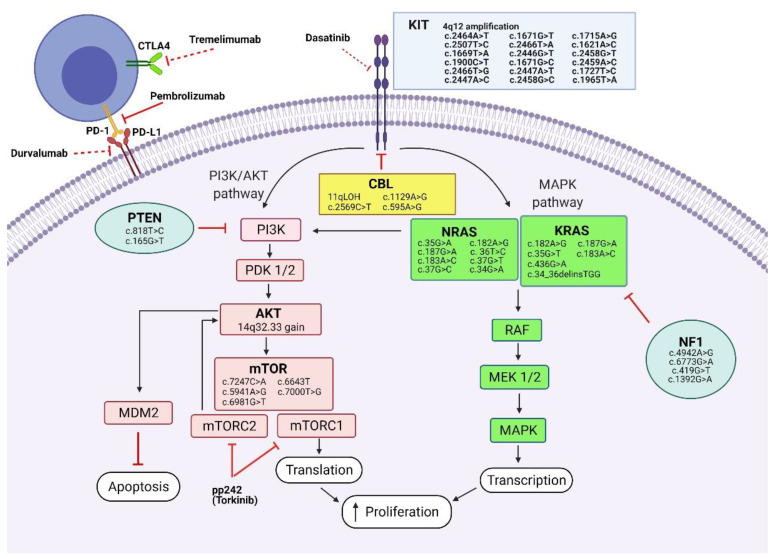
(Created with BioRender.com) Signalling pathways, genetic mutations and potential targeted treatment involved in CNS germinoma development. Tumourigenesis in germinoma is stimulated through various mechanisms. Activating KIT mutations promote cell proliferation via *PI3K/AKT* and *MAPK* pathways. Molecular aberrations involving the *PI3K/AKT* pathway result in increased proliferation via mTORC1 and cell survival by apoptosis inhibition via mTORC2. *MAPK* pathway mutations promote cell proliferation. Potential targeted treatment (KIT inhibitors, immune checkpoint inhibitors, mTOR inhibitors) are presented. Gene mutations identified in CNS germinomas are depicted alongside each gene. Abbreviations: KIT: tyrosine kinase receptor; CBL: casitas B-lineage lymphoma; KRAS: Kirsten rat sarcoma viral oncogene homologue; NRAS: neuroblastoma RAS viral oncogene homolog; RAF: rapidly accelerated fibrosarcoma kinase; MEK: mitogen-activated protein/extracellular signal-regulated kinase kinase; MAPK: mitogen-activated protein kinase; PI3K: phosphatidylinositol-3-kinase; PDK: pyruvate dehydrogenase kinase; AKT: v-akt murine thymoma viral oncogene homolog; mTOR: the mechanistic target of rapamycin; mTORC—mTOR complex; PD-1: programmed cell death protein 1; PD-L1: programmed death-ligand 1; CTLA4: cytotoxic T-lymphocyte-associated protein 4.

Gain of function mutations of *KIT* proto-oncogene generate a constitutive activation of the KIT protein, which consequently activates signal transduction molecules via *MAPK* (mitogen-activated protein kinase) or *PI3K* (phosphoinositide 3-kinase) pathways, resulting in increased cell proliferation, migration, and apoptosis resistance (Figure 5) [86]. 

However, so far, no correlations have been established between the *KIT* gene mutation and the expression of the KIT protein or clinical parameters (tumour location, size, and prognosis) [90,91]. The frequency and type of *KIT* mutation are differently distributed in the population: 5.9% in the germinomas encountered in the Chinese population (missense mutation in exon 11), whereas 23–25% in the Japanese patients (75% affecting the exon 17 and 25% the exon 11, others involving exons 2, 13) [89,90,91]. Another study mentions exon 10 variant (c.1621A>C) as the most common encountered in germinomas, other *KIT* exonic variants including exon 2 (c.251C>T), exon 11 (c.1658A>G), exon 13 (c.1965T>A), exon 17 (c.2447A>T) [92]. Nevertheless, 27.4% of CNS GCT have no detectable *KIT* mutations, even though KIT expression by IHC is high, suggesting implication of other mechanisms [88]. For example, *CBL* is a tumour suppressor gene involved in the process of down-regulation of the KIT receptor, and its mutation causes sustained KIT activation. Somatic mutations in the *CBL* gene are frequently encountered in ICGCT and represent another cause of KIT overexpression in germinomas [87,88]. 

The *PI3K/AKT* and *MAPK* pathways seem to be widely implicated in the pathogenesis of germinoma, being present simultaneously in 83% of tumour cells [14]. *MAPK* pathway alterations are more frequent in germinomas than NGGCT and have a tendency to correlate with a better prognosis, in comparison with *PI3K* pathway mutations. Upregulation of *MAPK* pathway by somatic point mutations represents the dominant genetic alteration in germinomas (64.3% of cases) [88]. These cases are more frequent in male patients and seem to be associated with an elevated serum HCG [7]. A case of 16p11.2 microdeletion was associated with the presence of bifocal germinoma, presumably due to deletion of *MAPK3* gene [93]. Amplification of 12p involves the *KRAS* gene (a component of *MAPK* pathway) and seems to be playing an important role in germinomas, similar to testicular germ cell tumours [14]. Furthermore, *NF-1*, a negative regulator of *MAPK* pathway, can present mutations in both germinomas and NGGCT [88].

Upregulation of *PI3K* pathway is the second genetic event involved in germinoma pathogenesis, *MTOR* gene being frequently mutated [88]. *MTOR* mutation promotes cell proliferation via *mTORC1* and cell survival via *mTORC2* and *AKT*. These effects were downregulated by pp242, an MTOR inhibitor, underlining the therapeutic prospects in germinoma [88]. Basal ganglia germinomas appear to frequently present *PI3K/mTOR* pathway mutations and chromosomal losses (1p, 3p/q, 4p, 9p/q, 10p/q, 11p, 13q, 18p/q, 19p/q, and 20p) [7]. Therefore, blockade therapy targeting these pathways may represent an alternative for germinomas that fail to respond to the standard regimens.

Compared to NGGCT, germinomas present with overexpression of genes within 4q13.3–4q28.3 and genes involved in self-renewal mechanisms (see Table 3) that have the capacity to induce dedifferentiation of matured somatic cells to pluripotent embryonic stem cells [94]. Takayasu et al. suggest that gene mutation analysis using CSF circulating tumour DNA is also a feasible study method in germinomas [95]. Genes reported in at least two distinct studies, and their genetic alterations are presented in Table 5. 

Recently, microRNAs (miRNAs) have been evaluated as possible biomarkers for various pathologies. MiRNAs are small, noncoding RNA molecules, involved in post-transcriptional gene regulation. GCT are associated with up-regulation of the miR-371~373 and miR-302 clusters, disregarding tumour site, age, or histopathologic type [96]. MiR-371a-3p was identified as a reliable marker in the differential diagnosis between germinoma and Langerhans cell histiocytosis, granting an early detection in cases where imaging studies and serum/CSF work-up are inconclusive [97]. A prospective observational cohort study is currently recruiting patients to evaluate whether miRNA 371 can be used as a prognostic marker for the risk of GCT recurrence [98]. MiR-142-5p and miR-146a are upregulated in the paediatric CNS germinoma, the former inversely correlating with *NRP1* (Neuropilin 1), *SVIL* (Supervillin), and *PDGFRA* (Platelet Derived Growth Factor Receptor Alpha) and the latter with *RUNX1T1* (RUNX1 Partner Transcriptional Co-Repressor 1) and *THRB* (Thyroid Hormone Receptor Beta) [94]. Low et al. observed a persistent correlation between *KIT* and downregulation of MiR-221-3p, although further studies are needed to validate this association and evaluate its clinical implications. Downregulation of miR-503 is also encountered in germinomas (Table 6) [92].

## 6. Immunological Approach 

Other factors seem to be involved in the pathophysiology of germinomas, tumour immune microenvironment being one of them [73]. Paradoxically, the large immune infiltrate encountered in germinomas seems to have no antitumour effect, and several studies aimed to evaluate the role of the programmed death receptor 1 (PD-1)/programmed death receptor 1 ligand (PD-L1) pathway, after it has been reported that PD-L1 is expressed by testicular seminomas, the gonadal counterpart to CNS germinomas [100]. The interaction between the ligand PD-L1, expressed by tumour cells, and PD-1 localised on activated lymphocytes induces T-cell anergy and downregulates the immune response. A small study of 8 patients with intracranial germinoma showed in all patients PD-L1 staining of the tumour cells and PD-1 expression of the tumour-infiltrating lymphocytes (TILs) and found a correlation between the immunosuppressive microenvironment and the growth of the tumours [101]. Takami et al. evaluated the tumour immune microenvironment in 32 germinoma cases, concluding that tumour cells have a 73.5% positivity for PD-L1, while the majority of infiltrating, stained immune cells are PD-1 positive (93.8%), making germinomas a proper candidate for immunotherapy [102]. The high expression of CD4 T helper lymphocytes correlates with a good prognosis, while great levels of nitric oxide synthase 2 produced by myeloid-derived suppressor cells and macrophages associates with a shorter progression free survival (PFS), possibly by generating immune tolerance [102]. Although there are some discrepancies between studies (Table 7), the role of PD-1/PD-L1 pathway in germinomas warrants further investigation and may offer new potential therapeutic perspectives.

## 7. Current Management 

The treatment of intracranial germinomas is multidisciplinary, including surgery, chemotherapy, radiotherapy (RT), and endocrine therapy. Germinomas are distinctively radiosensitive, with overall survival rates of over 90%, with radiation therapy alone [6,22,105,106]. Chemotherapy alone can induce complete remissions in 84% of the cases, but the long-term efficacy has been proven to be unsatisfactory, with high rates of morbidity and mortality, only 50% of the patients being treated successfully by this method [107,108,109]. Therefore, the standard treatment is represented by a combination of chemotherapy (Carboplatin/Cisplatin and Etoposide ± Ifosfamide) and radiotherapy [110,111]. Nevertheless, surgical resection is commonly performed as the first therapeutic option. The largest multicentre analysis of pituitary germinomas (SEER—Surveillance, Epidemiology, and End Result program) showed that chemotherapy was used more frequently in paediatrics, whereas surgery being applied in the adult population [112].

However, given the high radiosensitivity of germinomas, the extensive tumour resection is not necessary for a complete response, the surgery approach being currently limited to the treatment of hydrocephalus and a tumour biopsy in order to obtain a histological diagnosis. Obstructive hydrocephalus represents a severe complication and requires appropriate management. Until recently, increased intracranial pressure was relieved by ventriculoperitoneal shunts. However, it was discovered that this technique involves a high risk of peritoneal metastasis of the primary GCT and that patients require a thorough follow-up with CT scans of the abdomen to detect metastatic disease [113]. Nowadays, hydrocephalus can be treated by performing endoscopic ventriculostomy, without the risk of peritoneal metastasis [37]. Different techniques have been proposed for the approach, combining third ventriculostomy (ETV) with endoscopic biopsy. Usually, posterior third ventricle tumours are approachable through two trajectories, necessitating two burr holes or one “compromised” burr hole. Roth et al. performed this combined technique through a single burr hole, using a rigid endoscope to perform the ETV, followed by a flexible one to retrieve the biopsy sample, with favourable results. The benefits of this technique are represented by the necessity of a single burr hole, a better visualization of the tumour offered by the rigid endoscope, and a better access offered by the flexible one [114]. Moreover, a combined intervention for pineal tumours, using a single burr hole and a rigid endoscope for both ETV and biopsy, was successfully performed. The larger forceps of the rigid endoscope have the advantage of obtaining larger samples in comparison with flexible endoscopes [71,115]. Supracerebellar infratentorial approach, performed by microsurgery or endoscopic techniques, is a feasible alternative in cases of pineal tumours. The endoscopic approach offers a better visualization of the tumour, a wider range of motion of surgical instruments, a shorter duration of the procedure, a quick recovery, and fewer complications [116,117]. Second-look surgery should be considered in patients with residual tumour or high serum/CSF markers after an appropriate treatment protocol. In these instances, it is possible that the tissue sample was insufficient, containing only the germinomatous component of a tumour with a mixed histology [118,119].

The treatment for localized disease may consist of either craniospinal irradiation (CSI) alone, or chemotherapy and reduced-field radiotherapy. Whole ventricular irradiation (WVI) is recommended, as the ventricles and periventricular areas represent a frequent site of relapse [76,120]. Most studies recommend chemoradiotherapy (CRT), to avoid whole brain irradiation (WBI) and CSI and the adverse effects of radiotherapy. In addition, Zhang et al. suggest that limited radiotherapy represents a feasible treatment strategy for bifocal germinomas, without metastasis, as well [121]. This method of treatment ensured the achievement of long-term survival rates as high as 95–97% [122,123]. In addition, a prospective multicentre cohort study revealed that relapse rates could be reduced by adapting the RT volume as follows: whole ventricle radiotherapy (WVRT) for localized pineal/suprasellar lesions, whole-brain radiotherapy (WBRT) for localized basal ganglia/thalamus, and CSI for disseminated disease [124]. Moreover, in an analysis of 253 cases of CNS germinomas, Jennings et al. reported that signs and symptoms suggestive of hypothalamic-pituitary axis dysfunction should steer the treatment towards CSI and systemic chemotherapy [125]. Optimal RT dosage and field inclusion are still under debate. Selected prospective studies evaluating different treatment regimens (doses of chemotherapy and radiotherapy), and their conclusions are summarized in Table 8, and retrospective studies are resumed in Appendix A Table A1. We conducted a PubMed search for English written articles regarding intracranial germinomas, published between 1996–2021. The articles were selected based on a combination of search terms (germinoma, intracranial, CNS, germ cell tumour, pineal, suprasellar, bifocal, paediatric, treatment, radiotherapy, chemotherapy). We analysed studies evaluating solely germinomas, as well as studies evaluating a wider range of germ cell tumours, provided germinomas were included. 

As far as the radiotherapy techniques are concerned, proton beam therapy (passively scattered proton therapy and spot scanning proton therapy) delivers lower doses of radiation to the healthy tissue surrounding the tumour, sparing greater volumes of the temporal lobes and hippocampus than intensity-modulated radiotherapy (IMRT) [126].

**Table 8:** Prospective studies regarding treatment regimes in CNS germinomas.

Study	Chemotherapy Regimen ± Surgery	Radiotherapy	Results	Conclusion
**Lee et al. 2019 [124]** Prospective, multicentre, cohort study Bifocal germinomas treated as disseminated disease 91 germinoma patients: 65 localized diseases 9 bifocal diseases 17 multiple/disseminated cases Median age = 14 years 74.7% male patients	**±Surgery:** total/partial resection (11%)/biopsy (89%) 2 courses of: Etoposide 150 mg/m^2^ Carboplatin 450 mg/m^2^ alternating with 2 courses of: Etoposide 150 mg/m^2^ Cyclophosphamide 1000 mg/m^2^	**Localized disease:** CR: focal RT (30.6 Gy) PR: CSI (19.5 Gy) + focal RT (19.8 Gy) **Bifocal/multiple/disseminated disease:** CR: CSI (19.5 Gy) + focal RT (10.8 Gy) PR: CSI (24 Gy) + focal RT (16.2 Gy)	Median FU = 5.6 years 4 patients with progression/recurrence (4.4%) 5-year OS = 98.8% 5-year PFS = 96.6%	RT for localized pineal/suprasellar germinomas should include the whole ventricle area, whereas basal ganglia/thalamus germinomas should be treated with WBRT.
**Calaminus et al. SIOP 96, 2013 [76]** Prospective, multinational, nonrandomized study 235 germinoma patients: 190 localized diseases: 93 pineal; 53 supra-/intrasellar; 32 bifocal; 11 other 45 disseminated cases: 17 pineal, 13 supra-/intrasellar, 15 bifocal Median age = 13 years 176 males 59 females	**±Surgery:** 22 complete resections, 107 subtotal resections/open biopsies, 103 stereotactic biopsies **Local/bifocal disease** Carboplatin 600 mg/m^2^/day+ Etoposide 100 mg/m^2^/day alternating with Etoposide 100 mg/m^2^/day+ Ifosfamide 1800 mg/m^2^/day	**Local/bifocal disease:** Focal RT (40 Gy)	Median FU = 6 years 5-year PFS = 88% OS = 96% 7 recurrences (6 with ventricular relapse)	Ventricular relapses suggest the importance of WVRT Local/bifocal germinomas are successfully treated with reduced dose CSI or with chemotherapy and reduced field RT
	No chemotherapy	CSI 24 Gy + primary tumour site boost 16 Gy	5-year PFS = 97% OS = 95% 4 recurrences at original site	
	**Disseminated disease** ±Chemotherapy (same regimen)	**Disseminated disease** 24 Gy CSI +16 Gy boost at primary tumour site and metastases	5-year PFS = 100% OS = 98%	
**Kretschmar et al. POG 2007 [127]** Prospective, phase II study 12 germinomas: 8 localized diseases: 2 pineal, 2 basal ganglia, 1 suprasellar, 3 other 4 disseminated cases Median age = 15.1 years 10 male patients, 2 female patients	±Surgery: 5/12 patients: partial/total resection 4 courses Cisplatin 20 mg/m^2^/day+ Etoposide 100 mg/m^2^/day alternating with Vincristine 1.5 mg/m^2^ + Cyclophosphamide (CPM) 1 g/m^2^/day	CR: primary site RT 30.6 Gy; PR: primary site RT 50.4 Gy with 2 cm margin (3D-CRT) or 0.5 cm margin (SRT) **Disseminated disease:** CR: CSI 30.6 Gy + local boost 50.4 Gy PR: CSI 36 Gy + local boost 54 Gy	Median FU: 5.5 years 11/12 progression-free at median 5.5 years 1/12 refused RT, recurred at 10 months, salvage CSI, progression-free at 4.8 years	Favourable response (91%) and survival in the setting of chemotherapy followed by response-based RT
**Aoyama et al. 2002 [118]** Prospective study, phase II 27 germinomas out of 33 ICGCT: **16 pure germinomas** 8 localized diseases: 1 neurohypophysis, 7 pineal 6 multifocal diseases: 4 neurohypophysis + pineal ± ventricle, 1 neurohypophysis + ventricle, 1 bilateral basal ganglia 2 disseminated cases **11 β-HCG secreting germinoma** 8 localized diseases: 6 neurohypophysis, 2 pineal 2 multifocal diseases: neurohypophysis + pineal 1 disseminated case Mean age = 15.9 years (including patients with NGGCT) 32 males, 1 female (including patients with NGGCT)	±**Surgery**—3 gross total/3 partial resections, 10 biopsies for **pure germinomas**; 1 gross total/3 partial resections, 7 biopsies for 11 **β-HCG secreting germinoma** **Localised disease:** Etoposide (100 mg/m^2^) + Cisplatin (20 mg/m^2^) 5 consecutive days every 4 weeks −4 cycles after partial resection/biopsy −3 cycles after total/subtotal resection	**Localised disease:** Local RT 24 Gy	Mean FU = 4.8 years **Pure germinomas:** CR = 100% 5-year FSR = 90% 1 recurrence	WV or larger field irradiation (probably 30–40 Gy) is necessary for β-HCG secreting germinomas 24-Gy irradiation to the primary site in combination with EP chemotherapy yielded excellent results in solitary pure germinomas
	**Multifocal/disseminated disease + β-HCG-secreting germinomas:** 3–6 cycles of ICE: Ifosfamide (900 mg/m^2^) + Cisplatin (20 mg/m^2^) + Etoposide (60 mg/m^2^) 5 consecutive days every 4 weeks. **Recurrence:** chemotherapy and reirradiation	**β-HCG secreting germinomas:** Local irradiation/ 24-Gy WVRT+ 6 Gy neurohypophysis + 10 Gy pineal region **Multifocal:** 24 Gy WVRT **Disseminated:** 24 Gy CSI	**β-HCG-secreting** **germinomas:** CR = 100% 5-year free survival rate = 44% 5 recurrences No death due to recurrence	
**Matsutani, M. and The Japanese Pediatric Brain Tumor Study Group 2001 [128]** Prospective, phase II study 75 germinomas 10 germinomas with STGC	**Pure germinoma:** 8 total resections 3 courses PE: Cisplatin 20 mg/m^2^ + Etoposide 60 mg/m^2^ (days 1–5) or CARB-VP: Carboplatin 450 mg/m^2^ (day 1) + Etoposide 150 mg/m^2^ (days 1–3). Large tumours or multiple/disseminated tumours: 3 cycles of ICE: Ifosphamide 900 mg/m^2^ + Cisplatin 20 mg/m^2^ + Etoposide 60 mg/m^2^ (days 1–5)	**Pure germinoma** Local RT 24 Gy	**Pure germinoma** Median FU = 2.9 years CR = 92% Recurrence = 9 (12%), of which 7 outside of the irradiated area	WBRT is not necessary for germinomas A dose of 24 Gy to the primary tumour site is suitable for obtaining disease control
	**Germinoma with STGC:** 1 total resection CARB-VP or PE followed by RT+ same chemotherapy as RT for a total of 5 courses	**Germinoma with STGC** 30 Gy to a generous local area + 20 Gy to the primary tumour site	**Germinoma with STGC** CR = 90% No recurrences	
**Bamberg et al. MAKEI 83/86/89, 1999 [22]** Prospective, multicentre, non-randomized 60 germinomas: 26 pineal, 9 suprasellar, 7 bifocal, 11 multiple midline tumours, 7 frontal horns/lateral ventricles germinomas Median age = 13 years 45 males, 15 females	**±Surgery:** 5 complete resections 25 incomplete resections 4 open biopsies 24 stereotactic biopsies **±Chemotherapy** (salvation treatment)	MAKEI 83/86: 11 germinomas CSI 36 Gy + local boost 14 Gy (total 50 Gy)	Median FU = 9.8 years 5-year RFS = 100% OS = 100%	Dose reduction of RT is possible with reasonable results
		MAKEI 89: 49 germinomas 30 Gy (CSI) + local boost 15 Gy (total 34 Gy)	Median FU = 5.1 years 5-year RFS = 89% OS = 92%	
**Bouffet et al. SFOP- 1999 [129]** Prospective, multicentre study 57 germinomas: 20 pineal, 28 suprasellar, 2 thalamic, 7 bifocal, 6 disseminated cases Median age = 13.5 years 43 males, 14 females	**± Surgery:** 6 total resections, 12 partial resections, 7 open biopsies, 22 stereotactic biopsies 4 courses: Carboplatin 600 mg/m^2^ (day 1)+ Etoposide 150 mg/m^2^/day (days 1 to 3) alternating with Ifosfamide 1.8 g/m^2^/day (days 21 to 25) + Etoposide 150 mg/m^2^/day (days 21 to 23)	**Local/bifocal germinoma:** 40 Gy RT at primary tumour site **Disseminated germinoma:** CSI 25–30 Gy + 10 Gy boost on metastasis	Median FU = 3.5 years 3-year RFS = 98% 3-year EFS = 96.4% Recurrence: 4 (3 in second complete remission after salvage chemotherapy ± CSI)	Combination treatment with chemotherapy and local RT yielded favourable survival rates in local and bifocal germinoma
**Sawamura et al. 1998 [130]** Prospective, multicentre study **12 pure germinomas:** 4 pineal, 2 neurohypophyseal, 4 multifocal, 2 disseminated **5 β-HCG secreting germinomas:** 1 pineal, 3 neurohypophyseal, 1 disseminated Median age = 17 years 16 males, 1 female	**Surgery:** 3 total resections, 2 subtotal resections, 2 partial resections, 10 biopsies **Local:** 3/4 cycles EP: Cisplatin 20 mg/m^2^/day + Etoposide 100 mg/m^2^/day 5 consecutive days	**Local disease:** 24 Gy at primary tumour site	Median FU = 2 years 2-year survival probability = 100% 16 (94%) of the 17 patients were free from recurrence 1 recurrence—currently in second complete remission	Chemotherapy (EP and ICE regimens) followed by reduced volume and dose RT were highly effective in obtaining disease control
	**Multifocal, disseminated, β-HCG secreting germinoma:** 3–6 cycles ICE: Ifosfamide 900 mg/m^2^/day+ Cisplatin 20 mg/m^2^/day+ Etoposide 60 mg/m^2^/day 5 consecutive days **Recurrent disease**—same as disseminated	**Multifocal disease:** 24 Gy at primary tumour site **Disseminated disease:** CSI 24 Gy		
**Balmaceda et al. 1996 [109]** Prospective study 45 germinomas out of 71 ICGCT: 31 pineal, 21 suprasellar, 11 bifocal, 8 other, including 11 leptomeningeal disease Median age = 12.7 years (out of 71 ICGCT) 51 males, 20 females (out of 71 ICGCT)	±**Surgery:** 18 total resections, 18 partial resections, 33 biopsies (out of 71 ICGCT) 4 cycles of: Carboplatin 500 mg/m^2^/day days 1–2+ Etoposide 150 mg/m^2^/day days 1–3+ Bleomycin 15 mg/m^2^/day day 3 If CR: 2 more cycles If PR: 2 more cycles + cyclophosphamide ± RT	RT—if less than CR after chemotherapy or progressive/relapsed disease	Median FU: 2.9 years CR = 82% 2-year OS = 84% 20 relapses/45 germinomas	Treatment with chemotherapy alone is characterized by a high rate of tumour recurrence

Abbreviations: CR = complete remission, PR = partial remission, RT = radiotherapy, CSI = craniospinal irradiation, FU = follow-up, OS = overall survival, PFS = progression free survival, WBRT = whole-brain radiotherapy, SIOP = Société Internationale d’Oncologie Pédiatrique, WVRT = whole-ventricle radiotherapy, POG = Pediatric Oncology Group, ICGCT = intracranial germ cell tumour, CPM = cyclophosphamide, 3D-CRT = conventional planning radiotherapy, SRT = stereotactic radiotherapy, WV = whole ventricle, NGGCT = non-germinomatous germ cell tumour, β-HCG = β-human chorionic gonadotropin, ICE = ifosfamide + cisplatin + etoposide, STGC = syncytiotrophoblastic giant cells, EP/PE = etoposide + cisplatin, CARB-VP: carboplatin + etoposide, MAKEI = Maligue Keimzelltümoren, SFOP = Société Française d’Oncologie Pédiatrique, RFS = relapse free survival, EFS = event free survival.

Moreover, there appears to be a correlation between the ventricles volume and the dose of irradiation received by the healthy brain tissue (at least 12 Gy) after WVI and the boost phase of the treatment with IMRT (the smaller the ventricles, the smaller the dose of irradiation of the brain) [131]. Recently, gamma knife radiosurgery has been proposed as an integrated therapy in the management of pineal tumours. As far as germinomas are concerned, stereotactic radiosurgery (SRS) seems to be an effective boost local treatment that warrants the administration of a smaller dose of CSI, in order to minimize the adverse effects in the long-term, as well as a suitable treatment for tumour recurrence. Pineal germinomas treated with SRS alongside with standard adjuvant treatment showed a 20-year local control rate and survival of 80% [132]. Patients with metastatic pineal germinoma have a bad prognosis, with a 65.8% rate of long term survival and an increased risk of death represented by diffuse cerebral subarachnoid or leptomeningeal dissemination [26].

The principle of radiotherapy is to irradiate all the tumour cells disseminated in the ventricular system, by covering the cerebrospinal fluid pathway sufficiently. However, whole ventricle and boost irradiation of the tumours situated in the midline structures of the brain also deliver a heavy dose of radiation to the temporal lobes and the hippocampus, structures involved in learning and memory, causing neuropsychological deficits and endocrine dysfunctions [126,129,133,134,135]. These are frequently encountered in high-dose regimens, suggesting that strategies that limit cranial irradiation and the use of simultaneous integrated boost techniques may reduce the adverse effects associated with radiotherapy, having a beneficial impact on the quality of life of the patients [136,137,138,139]. A retrospective multi-centre cohort study evaluated the long-term toxicity of the treatment in 112 ICGCT, including 94 germinoma patients. Neurocognitive dysfunction was the most common adverse effect, while suprasellar/hypothalamic tumours and cisplatin treatment were associated with a high risk of hypopituitarism (36.1%) and ototoxicity (39.2%) respectively [140]. Hypopituitarism induced by radiotherapy is encountered in 90% of the cases after radiation treatment of the germinomas located in the neurohypophyseal region, but it also develops after treatment of germinomas located in other sites of the CNS [33]. However, a recent retrospective study, including 49 intracranial germinomas, concluded that hypopituitarism is mainly caused by the tumour and that radiotherapy has no further impairment on the pituitary function [141].

Restoration of endocrine deficiencies has been described in patients treated with chemotherapy alone, suggesting that delaying radiotherapy, especially in young patients with germinomas, may represent a treatment modality [142,143]. With early diagnosis of hypopituitarism and a suitable hormone replacement therapy, most patients achieve an average adult height [21]. Preoperative DI represents a positive predictor factor for the necessity of postoperative long-term hormonal replacement with desmopressin [144]. Secondary malignancies, such as glioblastoma, meningioma, thyroid carcinoma, acute lymphoblastic/myeloid leukaemia, and B-cell lymphoma, have been reported, highlighting the necessity of vigilant follow-up [145,146]. 

The patient follow-up consists in regular clinical evaluations (neurologic, endocrine, visual, and hearing assessments), tumour markers (serum and/or CSF AFP and β-HCG), and MRI scans. Tumour markers and imaging examinations should be evaluated simultaneously, since the diagnosis of relapsed disease can be established either on the presence of elevated markers or on the appearance of a new lesion [129,147]. Currently, there is no consensus regarding the superior sensitivity between serum and CSF markers. Initial evaluation shall be carried out 1–2 months after treatment completion and every 4–6 months thereafter, for the first 2–5 years, followed by annual assessments [51,76,148,149].

## 8. Future Perspectives

Ongoing molecular research aims to shed light upon germinoma’s underlying genomic and epigenetic mechanisms and their impact on the tumour evolution and treatment response. In this regard, molecular targeted therapy, such as selective tyrosine kinase inhibitors (TKI), may achieve promising results in cases of intracranial germinomas presenting KIT mutations that are resistant to standard CRT. Treatment with TKI has been proposed, considering their efficiency in cases of gastrointestinal stromal tumours (GIST) harbouring *KIT* mutations. Imatinib, a selective TKI, showed efficacy in cases of GISTs resistant to conventional treatment that presented exon 9 and 11 mutation [150]. Similarly, in CNS germinomas, *KIT* exons 11 and 17 are most frequently mutated, followed by exons [2,10,13,14,87,89,90,92]. Therefore, it can be presumed that germinomas harbouring exon 11 mutations are candidates for Imatinib therapy. *KIT* mutation V560D (Val560Asp), reported in one case of CNS germinoma, is likely to respond to Imatinib, as was previously reported in cases of GIST and mastocytosis harbouring the same mutation [89]. Ripretinib, another *KIT* inhibitor, is effective in cases of Imatinib resistant GISTs presenting D816V (Asp816Val), a mutation in exon 17 also encountered in CNS germinomas [151]. Furthermore, Dasatinib, a tyrosine kinase inhibitor (TKI) that crosses the blood–brain barrier, was evaluated in a retrospective review including five patients with CNS pure germinomas. However, despite the multimodal treatment, four patients finally experienced disease progression, and the efficacy of Dasatinib was not demonstrated [152]. Nevertheless, an ongoing phase I/II trial (NCT00788125) evaluates the treatment response to Dasatinib in combination with Ifosfamide, Carboplatin, and Etoposide of various tumours, including extragonadal germ cell tumours [153]. 

Other therapeutic options include *MAPK* and *AKT*/*mTOR* inhibitors. Ichimura et al. evaluated on cell cultures the effect of pp242 (Torkinib), an inhibitor of both *MTOR* complexes (*mTORC1* and *mTORC2*), on two germinoma *MTOR* mutations (M2327I and L2334V). They discovered that Torkinib inhibits in a dose-dependent manner the phosphorylation, cell migration, and growth induced by the mutated *MTOR* [88]. These results confirm that the mutations analysed are pathogenic and suggest that Torkinib warrants further evaluation as a potential targeted molecular agent in resistant CNS germinomas. Targeted inhibition of *RAS*/*MAPK* pathway may play a role in recurrent or resistant tumours and warrants further investigation. 

Immune checkpoint blockade, with PD-1/PDL-1 inhibitors (Nivolumab, Pembrolizumab, Atezolizumab, Durvalumab), represents another potential alternative therapy for resistant cases, considering the presence of a large immune infiltrate and the reported high expressions of PD-1 and PD-L1. Zschäbitz et al. reported a partial response after 15 cycles of Pembrolizumab in a patient with metastatic pituitary germinoma that did not respond to Sunitinib and second-line chemotherapy [154]. A phase II study is currently recruiting patients to evaluate the response of resistant pineal germinomas to Durvalumab (PD-L1 inhibitor) in combination with Tremelimumab (CTLA-4 inhibitor) [155]. 

In order to select the most appropriate course of treatment, the patients require a personalized management. Relapsed/resistant cases may be enrolled in clinical trials evaluating targeted therapies in CNS germinomas. They should also benefit from genetic and immunological testing, to discover whether they present pathogenic mutations or express immunological markers. Based on these results, the patient may receive the appropriate treatment, in accordance with each genotype (tyrosine kinase inhibitors/*MTOR* inhibitors/immune checkpoint blockade). Considering the young age of the patients and the radiation side effects, it is also important to offer the best quality of life possible. In this regard, association between targeted therapy and standard CRT may permit dose reduction or even RT elimination.

## 9. Conclusions

Intracranial germinomas are rare tumours mainly affecting the paediatric population. Although CRT has favourable results, research has been carried out in the endeavour to minimize the RT dosage and field, to provide the best quality of life for these patients. Nevertheless, resistant/relapsed tumours are therapeutically challenging. These cases may require genetic and immunological testing to identify patients that may benefit from personalized targeted therapy. Ongoing clinical trials aim to evaluate the efficiency of molecular targeted therapy in these cases.

## Figures and Tables

**Table 1:** Clinical presentation.

Neurologic Symptoms	Endocrine Symptoms	Ophthalmologic Symptoms
headaches	diabetes insipidus	Parinaud’s syndrome
nausea	GH insufficiency	visual field deficits
projectile vomiting	hypogonadism	acuity deficits
papilledema	secondary hypothyroidism	
lethargy hemiparesis	hypocortisolaemia (secondary adrenal insufficiency)	
ataxia	menstrual irregularities	
	precocious puberty	

Abbreviations: GH: growth hormone.

**Table 2:** Immunohistochemical staining in pure germinomas and germinomas with STGC.

Staining	Location	Germinoma Cells	Syncytiotrophoblastic Cells
PLAP	Cytoplasm	+	−
C-kit	Membrane	+	−
OCT 3/4	Nucleus	+	−
HCG	Cytoplasm	−	+
AFP	Cytoplasm	−	−
CD30	Membrane	−	+
CK AE1/3	Cytoplasm	−	+
D2-40	Membrane	+	−
LIN28	Ribosomes	+	−
HPL	Cytoplasm	−	+
NANOG	Nucleus	+	−
ESRG	Nucleus	+	−
UTF1	Nucleus	+	−
SALL4	Nucleus	+	−

Abbreviations: STGC: syncytiotrophoblastic giant cells; PLAP: placental alkaline phosphatase; C-kit: transmembrane protein with tyrosine kinase activity (or CD117); OCT 3/4: octamer binding transcription factor 3/4; HCG: human chorionic gonadotropin; AFP: alpha fetoprotein; CD30: tumour necrosis factor receptor; CK AE1/3: cytokeratin AE1/3; D2-40: podoplanin; LIN28: RNA-binding protein LIN28; HPL: human placental lactogen; NANOG: transcription factor in embryonic stem cells; ESRG: embryonic stem cell-related gene protein; UTF1: undifferentiated embryonic cell transcription factor 1; SALL4: sal-like protein 4.

**Table 3:** Summary of basal genetic information in CNS germinomas.

Genetic Alteration	Genes/Chromosomes; Comments
DNA hypomethylation	Similarity to primordial cells; genomic instability
Chromosomal aberrations	
Gains	1q (56.7–65%), 2q, 4q, 7 (59%), 8 (67%), 11p, 12p (36.4–82%), 21q (63.6–76%) and × (53.5–72.7%)
Losses	1p/q, 3p/q, 4p, 5q (39%), 9q (39%), 9p, 10p/q, 11q (41–45.5%), 11p, 13q (45–53.3%), 15q, 16p (37%), 17q (36.4%), 18p/q, 19p/q, 20p
Syndromes	Association with Down, Klinefelter syndromes
Gene mutations	
KIT	Gain of function mutations
MAPK pathway	KRAS, NRAS, HRAS, RRAS2
PI3K pathway	AKT, MTOR
Overexpressed genes	Expressed at 4q13.3–4q28.3: DDIT4L, BANK1, CXCL9, CXCL11, HERC5, ELOVL6
Genes involving self-renewal mechanisms	POU5F1 (expressed at chr1q13.13), NANOG, DPP4, KLF4
Other genes	CBL, NF1, PTEN, BCORL1, NFE2L3, NFE2L3, HNRNPA2B1
MiRNA dysregulation	Proposed biomarkers for diagnosis and prognosis

Abbreviations: CNS: central nervous system, KIT: transmembrane protein with tyrosine kinase activity; MAPK: mitogen-activated protein kinase; KRAS: V-Ki-Ras2 Kirsten Rat Sarcoma 2 Viral Oncogene Homolog; NRAS: Neuroblastoma RAS Viral (V-Ras) Oncogene Homolog; HRAS: HRas Proto-Oncogene, GTPase; RRAS2: Ras-related protein R-Ras2; PI3K: phosphoinositide 3-kinase; AKT: v-akt murine thymoma viral oncogene homolog; MTOR: Mammalian Target Of Rapamycin; DDIT4L: DNA Damage Inducible Transcript 4 Like; BANK1: B Cell Scaffold Protein with Ankyrin Repeats 1; CXCL9: C-X-C Motif Chemokine Ligand 9; CXCL11: C-X-C Motif Chemokine Ligand 11; HERC5: HECT and RLD Domain Containing E3 Ubiquitin Protein Ligase 5; ELOVL6: ELOVL Fatty Acid Elongase 6; POU5F1: OCT4, POU Class 5 Homeobox 1; NANOG: Nanog Homeobox; DPP4: Dipeptidyl Peptidase 4; KLF4: Kruppel Like Factor 4; CBL: Casitas B-lineage Lymphoma; NF1: Neurofibromin 1; PTEN: Phosphatase and tensin homolog; BCORL1: BCL6 Corepressor Like 1; NFE2L3: Nuclear factor (erythroid-derived 2)—like 3; HNRNPA2B1: Heterogeneous nuclear ribonucleoprotein A2/B1.

**Table 4:** Frequency of *KIT* and *RAS* mutations in CNS germinoma.

Study	*KIT* Mutation	*RAS* Mutation
Schulte et al. [14]	17.3%	34.6%
Fukushima et al. [86]	40%	20%
Wang et al. [87]	24%	19%
Ichimura et al. [88]	40%	19%
Kamakura et al. [89]	23%	Not evaluated
Sakuma et al. [90]	25%	Not evaluated
Gao et al. [91]	5.9%	Not evaluated

Abbreviations: *KIT*: transmembrane protein with tyrosine kinase activity; *RAS*: Rat Sarcoma (oncogene); CNS: central nervous system.

**Table 5:** Genes with possible pathogenic role in germinomas, reported in at least two distinct studies.

Study	Chr.	Gene	AA Mutation	CDS Mutation
Ichimura et al. [88] Schulte et al. [14] Fukushima et al. [86] Wang et al. [87] Sakuma et al. [90] Takayasu et al. [95] Low et al. [92]	4q12	*KIT*	*p.Asn822Tyr* *p.Met836Thr* *p.557W>R* *p.Arg634Trp* *p.Asn822Lys* *p.Asp816Ala* *p.Asn655Lys* *p.820D>Y* *p.Asp572Gly* *p.Trp557Cys* *p.Asn822Lys* *p.Asp816Tyr* *p.Trp557Cys* *p.Asp816Val* *p.Asp820His* *p.Leu576Pro* *p.D820A* *p.M541L*	*c.2464A>T* *c.2507T>C* *c.1669T>A* *c.1900C>T* *c.2466T>G* *c.2447A>C* *c.1965T>A* *c.2458G>T* *c.1715A>G* *c.1671G>T* *c.2466T>A* *c.2446G>T* *c.1671G>C* *c.2447A>T* *c.2458G>C* *c.1727T>C* *c.2459A>C* *c.1621A>C*
			*+4q12 amplification*	
Ichimura et al. [88] Schulte et al. [14] Fukusima et al. [86] Wang et al. [87]	12p12.1	*KRAS*	*p.63E>K* *p.Gln61Arg* *p.Gly12Val* p.Gln61His p.146A>T p.G12W	*c.182A>G* *c.187G>A* *c.35G>T* c.183A>C c.436G>A c.34_36delinsTGG
			+12p12 amplification	
Ichimura et al. [88] Schulte et al. [14] Fukushima et al. [86] Wang et al. [87] Takayasu et al. [95]	1p13.2	*NRAS*	*p.12G>D* p.Glu63Lys p.Gln61His p.Gly13Arg p.Q61R p.G12B p.G13C p.G12S	*c.35G>A* c.187G>A c.183A>C c.37G>C c182A>G c.36T>C c.37G>T c.34G>A
Ichimura et al. [88] Fukushima et al. [86]	11p15.5	*HRAS*	*p.61Q>R*	*c.182A>G*
Ichimura et al. [88] Schulte et al. [14]	11p15.2	*RRAS2*	p.Gly23Cys p.G23A p.G23S p.G24C G24D	c.67G>T GGC → GCC GGC → AGC GGC → TGC GGC → GAC
			+11p15.2 amplification	
Ichimura et al. [88] Wang et al. [87]	1p36.22	*MTOR*	p.Ala2416Asp p.Lys1981Glu p.2327M>I p.2334L>V p.Ser2215Pro	c.7247C>A c.5941A>G c.6981G>T c.7000T>G c.6643T>C
Ichimura et al. [88] Wang et al. [87]	11q23.3	*CBL*	p.Leu857Phe p.Thr377Ala p.Ile199Val	c.2569C>T c.1129A>G c.595A>G
			+*11qLOH*	
Ichimura et al. [88] Schulte et al. [14]	17q11	*NF1*	p.Thr1648Ala p.Arg2258Gln p.Gly140Val	c.4942A>G c.6773G>A c.419G>T c.1392G>A (possible splice site disruption)
Ichimura et al. [88] Takami et al. [7]	10q23.31	*PTEN*	p.Phe273Ser p.Arg55Ser	c.818T>C c.165G>T
Ichimura et al. [88] Wang et al. [87]	Xq26.1	*BCORL1*	p.Thr853Asn	c.2558C>A c.1924Cdel c.4700AAdel
Schulte et al. [14] Wang et al. [94]	7p15.2-p14	*NFE2L3*	Chromosome number gains	
Ichimura et al. [88] Schulte et al. [14]	7p15.2	*HNRNPA2B1*	r.spl10 p.Ser212 * p.69F>S	INV10+1G>T c.635C>A c.206T>C c.878-1G>C (possible splice site disruption)

The genes showed in the table have been reported as mutated in CNS germinomas in at least two distinct studies. Only KIT mutations reported in at least two studies are presented. Other gene’s mutations validated in more than one study are written in italics. Mutations reported only once are written normally. Abbreviations: chr: chromosome; AA mutation: amino acid mutation (the change in the amino acid sequence caused by the mutation); CDS mutation: coding DNA sequence mutation (the change in the nucleotide sequence caused by the mutation); KIT: Receptor Tyrosine Kinase; KRAS: V-Ki-Ras2 Kirsten Rat Sarcoma 2 Viral Oncogene Homolog; *NRAS*: Neuroblastoma RAS Viral (V-Ras) Oncogene Homolog; *HRAS*: HRas Proto-Oncogene, GTPase; *RRAS2*: Ras-related protein R-Ras2; *MTOR*: Mammalian Target Of Rapamycin; *CBL*: Casitas B-lineage Lymphoma; *NF1*: Neurofibromin 1; *PTEN*: Phosphatase and tensin homolog; *BCORL1*: BCL6 Corepressor Like 1; *NFE2L3*: Nuclear factor (erythroid-derived 2)—like 3; *HNRNPA2B1*: Heterogeneous nuclear ribonucleoprotein A2/B1; the symbol “*” marks the stop codon.

**Table 6:** Up and downregulation of miRNAs in GCT.

Study	Upregulated	Downregulated
Murray et al. 2020 [97]	miR-371a-3p	
Low et al. 2020 [92]	miR-373-3p	miR-571
	miR-373-5p	miR-503-5p
	miR-455-5p	miR-324-5p
	miR-650	miR-221-3p
	miR-183-5p	miR-132-3p
Murray et al. 2016 [99]	miR–373–3p	
	miR–367–3p	
	miR–302a–3p	
	miR–302b–3p	
Wang et al. 2010 [94]	miR-146a	
	miR-142-5p	

Abbreviations: miRNA: microRNA; GCT: germ cell tumours.

**Table 7:** Germinoma immune microenvironment.

Study	No. Cases	PD-L1 Expression (Tumour Cells)	PD-1 Expression (Immune Cells)
Nishimoto et al. 2020 [101]	8	100%	100% TILs
Takami et al. 2019 [102]	32	73.5%	93.8% immune cells
Liu et al. 2018 [103]	25	92%	96% TILs
Wildeman et al. 2018 [104]	21	90%	48% lymphocytes, stromal cells
Zapka et al. 2017 [73]	28	0%	11.9% TILs

Abbreviations: no: number; PD-L1: programmed death receptor 1 ligand; PD-1: programmed death receptor 1; TILs: tumour-infiltrating lymphocytes.

## Appendix A

**Table A1:** Retrospective studies regarding treatment regimes in CNS germinomas.

Study	Chemotherapy Regimen ± Surgery	Radiotherapy	Results	Conclusion
**Lee et al. 2021 [156]** Retrospective study 189 germinomas: 119 solitary lesions: 46 pineal, 41 suprasellar, 29 basal ganglia/thalamus, 3 other 25 bifocal diseases 45 disseminated diseases Median age = 15 years 151 males, 38 females	No chemotherapy	CSI 24 Gy localised disease/30 Gy disseminated disease+ tumour boost 54 Gy	Median FU = 9.6 years **10-year OS:** RT only: 85.2% CRT: 92.8% **Relapse:** RT only: 10% CRT: 5.8% **Salvage treatment:** 50% response Hormone replacement correlated with WBRT Secondary malignancy: 5.3%, more frequent in WBRT, CSI	Chemotherapy permits the use of reduced RT dosage The dose and volume of extended-field RT should be reduced in order to prevent late adverse effects
	2 cycles: Bleomycin + Etoposide + Cisplatin/ Etoposide + Cisplatin OR 5 cycles Cisplatin + Etoposide + Cyclophosphamide + Vincristine OR “8-in-1”: Solumedrol + Vincristine+ Lomustine + Procarbazine + Hydroxyurea + Cisplatin + Cytosine Arabinoside + Cyclophosphamide	CSI 23 Gy + boost/ WBRT 23 Gy + boost/ WVRT 23 Gy + boost/ Focal RT Tumour boost dose: 45 Gy if CR after chemo 50 Gy if no CR after chemo		
	**Salvage therapy** after relapse: chemotherapy + CSI/chemotherapy only/CSI only			
**Nosrati et al. 2021 [157]** Retrospective study 4 localised germinomas out of 18 intracranial germinomas: 1 pineal, 2 suprasellar, 1 bifocal	4 cycles every 3 weeks: Carboplatin 300 mg/m^2^+ Etoposide 150 mg/m^2^	Simultaneous Integrated Boost (SIB): WVRT 22.5 Gy+ Tumour boost 30 Gy	SIB: EFS = 89.5% SIB: OS = 100% Organ-at-risk doses—comparable between SIB and WVRT only; lower for COG	SIB technique permits higher doses to primary tumour than WVRT only, without further neurocognitive impairment COG protocol–lower doses, but still evaluating efficiency
	1 cycle: Carboplatin 450 mg/m^2^+ Etoposide 150 mg/m^2^	WVRT 24 Gy		
	4 cycles every 3 weeks: Carboplatin 600 mg/m^2^+ Etoposide 150 mg/m^2^	Sequential therapy (COG protocol): WVRT 18 Gy+ Tumour boost 12 Gy		
**Baroni et al. 2021 [148]** Retrospective study 39 germinomas: 15 pineal, 9 sellar/suprasellar, 7 bifocal, 2 thalamus/basal ganglia, 1 other, 5 disseminated cases Median age = 12.1 years 27 males, 12 females	**Surgery:** 1 complete resection, 6 partial resections, 32 biopsies 2 courses of: Carboplatin + Etoposide alternating with Ifosfamide + Etoposide	FR/ FR + WVRT/ CSI only (1 patient)	Median FU = 6.5 years 5-year PFS = 83.5% 5-year OS = 88.7% 5-year PFS: FR: 63% FR + WVRT: 94% 4 deaths	FR increases the risk of recurrence Chemotherapy and focal RT + WVRT has favourable results in localized germinomas
	**1st line salvage therapy:** Platinum-based chemotherapy + CSI ± FR **2nd line salvage therapy:** 4 cycles: Gemcitabine-Paclitaxel-Oxaliplatin + HDC + stem cell rescue		7 localized germinoma relapsed (5 treated with FR/2 with FR + WVRT)	
**Li et al. 2021 [147]** Retrospective study 161 basal ganglia germinomas: 68 right, 66 left, 15 bilateral, 12 basal ganglia + sellar Median age = 12 years 150 males, 11 females	2 cycles every 4 weeks before and after RT: Ifosfamide, 1.5 g/m^2^ days 1–3+ Etoposide, 70 mg/m^2^ days 1–3+ Cisplatin, 30 mg/m^2^ days 1–3 **±Surgery:** 15 resection, 15 biopsies	FR/CSI (30 Gy) + tumour boost (10 Gy)/ WBRT (30 Gy) + tumour boost (10 Gy)	Median FU = 6.9 years 5-year DFS = 92% 5-year OS FR = 94.1% 5-year OS WBRT = 97.2% 5-year OS CSI = 100%	WBRT offers a better quality of life compared to CSI and represents an optimal treatment strategy in localized basal ganglia germinomas
**Kumar et al. 2021 [149]** Retrospective study 19 germinomas out of 28 ICGCT: 12 pineal, 3 suprasellar, 4 other Median age = 17 years (including NGGCT) 11 males, 8 females	**±Surgery:** 3 total resections, 4 subtotal resections, 7 biopsies, 9 shunts 4–6 cycles: Bleomycin + Etoposide + Cisplatin/ Etoposide + Cisplatin	Local RT/ WVRT + boost/ WBRT + boost/ **Disseminated:** CSI + boost 3D-CRT/IMRT Total dose: 36–40 Gy	Median FU = 4.4 years 5-year OS = 89% 5-year PFS = 83.1%	Chemotherapy and reduced dose/volume RT has favourable results
**Rajagopal et al. 2021 [158]** Retrospective study 16 germinomas: 6 pineal, 6 suprasellar, 1 bifocal, 2 basal ganglia, 1 other, including 7 disseminated cases Median age = 11 years 11 males, 5 females	**±Surgery:** 1 total resection, 3 subtotal resection, 12 biopsies, 3 ventriculoperitoneal shunts, 3 ventriculostomies SIOP CNS GCT 96 protocol/ SIOP CNS GCT II protocol/ physician preference	CSI (24–25 Gy) ± boost (40–44.4 Gy)/ WVRT (24–36 Gy) + boost (36–54 Gy)/ Local RT (40–50 Gy) **Recurrence:** Chemo + CSI 24 Gy + cranial RT 19.8 Gy	Median FU = 3.5 years 10-year OS = 75.5% 10-year EFS = 61.1% Recurrence: 5 patients	Inferior results are observed in underdeveloped countries due to late diagnosis, poor compliance, suboptimal treatment, and complications of therapy
**Hong et al. 2020 [146]** Retrospective study 66 germinomas + normal tumour markers out of 69 low-risk tumours: 24 pineal, 14 sellar/suprasellar, 17 thalamus/basal ganglia, 10 bifocal, 4 other, including 7 disseminated cases Median age = 11.8 years 55 males, 16 females	**±Surgery:** resection/biopsy/endoscopic third ventriculostomy/extraventricular drainage/ventriculoperitoneal shunt Carboplatin 450 mg/m^2^/day+ Etoposide 100 mg/m^2^/day Alternating with Cyclophosphamide 1000 mg/m^2^/day+ Etoposide 150 mg/m^2^/day	Local RT (40.4 Gy)/ WVRT (19.8 Gy)/ WBRT (30.6 Gy)/ CSI (23.4Gy)—multifocal/metastatic disease, poor response to chemotherapy	Median FU = 8.4 years 10-year EFS = 88.6% 10-year OS = 98.3% 10-year relapse incidence = 9.3% 4 basal ganglia germinoma relapsed Endocrine complications:53.6%	Chemo-radiotherapy achieved favourable results
14 germinomas + high tumour markers out of 58 high-risk tumours: 28 pineal, 13 sellar/suprasellar, 7 thalamus/basal ganglia, 7 bifocal, 3 other, including 7 disseminated cases Median age = 12.2 years 47 males, 11 females	Carboplatin 450 mg/m^2^/day+ Etoposide 150 mg/m^2^/day Bleomycin 15 mg/m^2^/day Alternating with Cyclophosphamide 2000 mg/m^2^/day+ Etoposide 150 mg/m^2^/day+ Bleomycin 15 mg/m^2^/day	WVRT (23.4 Gy)/ WBRT (30.6 Gy)/ CSI (23.4 Gy)	Median FU = 8.3 years 10-year EFS = 100% 10-year OS = 100% 10-year relapse incidence = 5.6% Endocrine complications:53.4%	Germinoma with elevated tumour markers could benefit from a lower intensity treatment
**Chou et al. 2020 [159]** Retrospective study 24 germinomas: 17 pineal, 13 suprasellar, 7 periventricular, 2 basal ganglia 13 isolated tumours 11 multifocal tumours 5 β-hCG-secreting germinomas Median age = 14.1 years 20 males, 4 females	**Surgery:** 1 total resection, 9 subtotal resections, 14 biopsies **Short-course** neoadjuvant chemotherapy: 2 courses of Cisplatin 20 mg/m^2^+ Etoposide 40 or 100 mg/m^2^ for 5 days	WVRT/WBRT (23.4 Gy) **without** local boost	Median FU = 8.8 years 100% complete remission 5-year DFS β-hCG-secreting germinomas = 60% 5-year DFS normal β-hCG level germinomas = 100%	CNS germinoma patients with normal β-hCG levels may benefit from short course chemotherapy and low dose RT without local boost
**Shimizu et al. 2020 [119]** Retrospective study 40 pure germinomas+ 30 germinomas with STGC out of 110 ICGCT: 52 pineal, 39 suprasellar, 16 basal ganglia, 10 other Median age = 14 years (including NGGCT) 90 males, 20 females (including NGGCT)	**±Surgery:** 1 total resection, 2 subtotal resections, 10 partial resections, 17 biopsies 2 courses before RT and 1 after: Carboplatin 300 mg/m^2^, day 1+ Etoposide 100 mg/m^2^, days 1–3 OR Chemotherapy only	WVRT (30 Gy) ± tumour boost (10 Gy) if remnant tumours/ **Basal ganglia germinoma:** WBRT (30 Gy)/ RT only	Median FU = 11 years 5-year OS = 97.1% 10-year OS = 95.7% 20-year OS = 93.2% 5-year PFS = 91.4% 10-year PFS = 86.6% 20-year PFS = 86.6% No neurocognitive disorders No treatment-related deaths	The CRT regimens presented yielded favourable results in pure germinomas and germinomas with STGC Further studies might attempt further reduction of RT dosage
	2 courses before RT and 1 after: Cisplatin 20 mg/m^2^ + Etoposide 60 mg/m^2^, days 1–5	Focal RT 40 Gy		
	**Multifocal/disseminated** Carboplatin 300 mg/m^2^, day 1+ Etoposide 100 mg/m^2^, days 1–3+ Ifosfamide 1500 mg/m^2^, days 1–3	**Multifocal/disseminated** CSI (30 Gy)		
**Esfahani et al. 2020 [60]** Retrospective study 33 germinomas: 15 suprasellar, 14 bifocal, 4 basal ganglia + suprasellar, including 12 disseminated cases Out of all 42 ICGCT: Mean age suprasellar tumours = 11.2 years Mean age bifocal tumours = 13.4 years 26 males,16 females	Neoadjuvant chemotherapy (COG protocol) **±Surgery:** resection/biopsy/second-look surgery, endoscopic/craniotomy/transsphenoidal/stereotactic	**Suprasellar germinomas:** WVRT + local boost /WBRT/Focal RT **Suprasellar germinomas + ventricular metastasis:** WVRT + local boost/ CSI + local boost **Bifocal germinomas ± ventricular metastases:** WVRT + local boost/ CSI + local boost **Basal ganglia germinoma** **±ventricular metastases:** CSI + local boost	**WVRT + boost:** 1 suprasellar germinoma recurrence 2 bifocal germinomas recurrences **CSI + boost:** 1 bifocal germinoma recurrence	Bifocal germinomas have a higher rate of metastasis and recurrence CSI is recommended for bifocal germinomas with metastasis
**Li et al. 2020 [160]** Retrospective study 49 non-metastatic bifocal germinomas: 34 sellar/suprasellar + pineal, 15 sellar/suprasellar + thalamus/basal ganglia Median age = 13 years 34 males, 15 females	Two courses before and after RT: Ifosfamide 1.5 g/m^2^ days 1–3+ Etoposide 70 mg/m^2^ days 1–3+ Cisplatin 30 mg/m^2^ days 1–3 **±Surgery**	CSI + boost/ WBRT + boost/ Focal RT	Median FU = 4.3 years 5-year DFS = 96.7% 5-year OS = 97.3% WBRT—comparable disease-free survival with CSI	Limited field RT could replace CSI in patients with non-metastatic bifocal germinoma
81 non-metastatic bifocal germinoma (literature): sellar/suprasellar + pineal	Not mentioned	Focal RT/WVRT/WBRT/CSI	No difference regarding the disease-free survival	
**Takada et al. 2018 [161]** Retrospective study 6 germinomas + 12 germinomas with STGC, out of 24 ICGCT: 10 pineal, 3 suprasellar, 6 basal ganglia/brainstem, 5 multifocal, 4 disseminated cases Median age = 13 years (including NGGCT) 20 males, 4 females (including NGGCT)	**Pure germinoma** **±Surgery:** 2 partial excisions, 2 biopsies 2–5 courses of conventional dose therapy (CDC): ICE: Ifosfamide + Cisplatin + Etoposide	WVRT 24 Gy	Median follow-up = 9.3 years 10-year OS = 100% 1 recurrence after 10 years	Favourable results obtained with combined treatment: chemotherapy (conventional/intensive) and reduced field and dose irradiation
	**Germinoma with STGC:** **±Surgery:** 1 total excision, 3 partial excisions, 4 biopsies 2/3 courses of CDC and high-dose chemotherapy (HDC) + PBSCT HDC: PEB/EP/CARE/ICE	WVRT/larger field 24–30 Gy ± local boost irradiation (20 Gy)	3 patients relapsed and died of their disease 10-year OS = 78.7%	
	No chemotherapy	CSI 24 Gy + primary tumour site boost 16 Gy	5-year PFS = 97% OS = 95% 4 recurrences at original site	
	**Disseminated disease** ±Chemotherapy (same regimen)	**Disseminated disease** 24 Gy CSI + 16 Gy boost at primary tumour site and metastases	5-year PFS = 100% OS = 98%	
**Cheng et al. 2016 [138]** Retrospective study 24 germinomas: 9 suprasellar, 6 pineal, 8 bifocal, 1 basal ganglia, including 4 disseminated cases Median age = 13.3 years 13 males, 11 females	**±Surgery:** biopsy, external ventricular drain, septostomy, ventriculostomy, ventricular-peritoneal shunt 2 cycles of Carboplatin 600 mg/m^2^ + Etoposide 300 mg/m^2^ alternating with Ifosfamide 9000 mg/m^2^ + Etoposide 300 mg/m^2^ OR 3–4 cycles Carboplatin 600 mg/m^2^ + Etoposide 450 mg/m^2^ 3–4 cycles	WVRT (23.4–24 Gy)/ WVRT (23.4–24 Gy) + boost (16 Gy)/ WBRT (23.4 Gy)/ Focal RT (40 Gy)/ CSI (23.4 Gy)	Median FU = 5 years 5-year PFS = 96% 5-year OS = 100% 1 recurrence No difference in neurocognitive functions between focal RT and WVRT	Successful results in the setting of CRT, with reduces dose and volume RT No relapses in cases of WVRT without boost
**Weksberg et al. 2012 [162]** Retrospective study 20 bifocal germinomas (pineal + suprasellar), including 11 disseminated diseases Median age = 19 years 19 males, 1 female	**±Surgery:** 13 biopsies ±Neoadjuvant platinum-based chemotherapy+ Etoposide± Ifosfamide Chemotherapy was not administered to patients who received CSI	Median RT dose = 50 Gy **Localized disease:** CSI/WBRT/WVI/local RT **Disseminated disease:** CSI/WBRT/WVRT	Median FU = 8.2 year 5-year PFS = 100%	Bifocal germinomas with no signs of dissemination are candidates for neoadjuvant chemotherapy and spinal sparing RT. CSI represents the treatment of choice for disseminated disease.
60 bifocal (pineal + suprasellar) germinomas (**literature**) including 14 disseminated diseases Age range: 6–30 years 43 males,11 females, 6 not stated	±Neoadjuvant platinum-based chemotherapy+ Etoposide± Ifosfamide/Methotrexate	Median RT dose = 40 Gy CSI/WBRT/WVI/local RT	Median FU = 5 years 5-year PFS = 87%	
**Combined analysis:** 80 bifocal germinoma (pineal+ suprasellar), including 25 disseminated cases 62 males, 12 females, 6 not stated Age range: 6–33 years	**55 localized diseases:** 5-year PFS = 95% 5-year PFS (CSI) = 100% 5-year PFS (WBRT/WVI /local RT) = 88% 5-year PFS (WBRT/WVI /local RT + Chemo) = 96%	**25 disseminated disease:** 5-year PFS = 80% 5-year PFS (CSI)= 100% 5-year PFS (WBRT/WVI /local RT + Chemo) = 69%		
**Macdonald et al. 2011 [163]** Retrospective study 13 germinoma patients: 7 suprasellar, 2 basal ganglia + thalamus, 4 multiple midline tumours Age range: 6–20 years 5 males, 8 females	**±Chemotherapy** cisplatin, carboplatin, etoposide, ifosphamide, cyclophosphamide, vincristine **±Surgery**	CSI (18–23 Gy) + involved field (5.4–36 Gy)/ WVRT (19.5–23.4 Gy) + involved field (7.2–22 Gy)/ WBRT (25.5 Gy) + involved field (19.8 Gy) * 3D PT compared to IMRT and IMPT	Median FU = 2.3 years Local control= 100% No CNS recurrences PFS = 95% OS = 100%	Favourable preliminary results Proton therapy provides superior dose-distribution, with greater normal tissue saving, compared to IMRT
**Eom et al. 2008 [164]** Retrospective study 81 germinoma patients: 22 suprasellar, 26 pineal, 13 suprasellar + pineal, 3 suprasellar + other, 17 other, including 21 disseminated cases Median age = 14.6 years (RT only group) Median age = 17.5 years (CRT group) 65 males, 16 females	**Surgery:** 27 stereotactic biopsies, 20 endoscopic biopsies, 10 open biopsies, 12 partial resections, 9 subtotal resections, 3 total resections **No chemotherapy**	CSI: 21 Gy—negative seeding 36 Gy—positive seeding +involved field 54 Gy	Median follow-up = 5.7 years No relapse 5-year OS = 100% 5-year RFS = 100%	Chemoradiotherapy provides better quality of live, but more relapses. Ventricles should be included in the RT field for the treatment of localized disease
	42 patients: chemo + RT 1–4 cycles of Bleomycin + Etoposide, and Cisplatin/Etoposide + Cisplatin **OR** 1–5 cycles of Cisplatin, Etoposide, Cyclophosphamide and Vincristine **OR** Solumedrol, Vincristine, Lomustine, Procarbazine, Hydroxyurea, Cisplatin, Cytosine arabinoside, and Cyclophosphamide	**Localised disease/negative** **seeding:** 50 Gy at tumour site **Multifocal disease/positive** **seeding:** CSI 23.4 Gy+ Local boost: 45 Gy—if CR after chemo 54 Gy—if PR after chemo	4 relapses in patients with involved-field RT 5-years RFS = 88% 5-year OS = 93%	
**Lafay-Cousin et al. 2006 [23]** Retrospective 6 patients with bifocal germinomas out of 17 germinomas: 4 pineal + suprasellar, 1 pineal + third ventricle floor, 1 pineal + tuber cinereum Median age = 12.8 years 5 males, 1 female	**±Surgery:** 3 third ventricle ventriculostomies + biopsies 3/4 cycles of Etoposide + Cisplatin OR 2 courses of Carboplatin + Etoposide alternating with 2 courses of Ifosfamide + Etoposide	WVRT 24–40 Gy ±boost at both tumour sites (16 Gy)	Median FU = 4 years CR = 100% No persistent disease/recurrence	Bifocal germinoma (pineal + suprasellar) can be treated as locoregional disease with chemotherapy and limited field radiation (WVRT ± boost)
**Modak et al. 2004 [165]** Retrospective 9 patients with relapsed germinoma out of 21 CNS relapsed GCT: 3 intracranial, 1 spinal cord, 1 intracranial + spinal cord, 1 ventricular, 1 pineal + abdominal, 1 pineal + lateral horn, 1 leptomeningeal dissemination Age range: 7–31 years 7 males, 2 females	**±Surgery:** 4 biopsies Thiotepa 200 mg/m^2^/d for 3 days with ASCR; followed 4 weeks later by Thiotepa 200 mg/m^2^/d for 3 days with ASCR Or Carboplatin 500 mg/m^2^/d on days 1–3 +Thiotepa 300 mg/m^2^/d on days 4–6 +Etoposide 250 mg/m^2^/d on days 4–6 Or Carboplatin 500 mg/m^2^/d on days 1–3 +Thiotepa 300 mg/m^2^/d on days 4–6 +Temozolomide 150–250 mg/m^2^/d	Post-chemotherapy adjuvant RT: −cranial irradiation −CSI ± focal irradiation	Median FU = 4 years OS = 78% PFS = 78% 2 deaths: −1 progressive disease (PD) −1 pulmonary fibrosis secondary to bleomycin and spinal irradiation	High dose chemotherapy followed by ASCR represents an effective alternative therapy for patients with relapsed CNS germinomas
**Haas-Kogan et al. 2003 [166]** Retrospective 49 germinomas: 41 localized 8 disseminated Median age = 15 years 32 males, 17 females	**±Surgery:** 22 biopsies, 19 subtotal resections, 5 total resections ±Chemotherapy	**Localised disease:** CSI/WBRT/WVRT (32.4 Gy) + local boost/Local RT **Disseminated disease:** CSI	Median FU = 7.5 years 5-year OS = 93% 5-year PFS = 88% 5 relapses 2 deaths	CSI is not necessary for localized disease; WVRT + local boost (total of 45–50 Gy) is recommended in localized disease
**Fouladi et al, 1998 [167]** Retrospective study 16 germinomas: 6 pineal, 3 suprasellar, 3 bifocal, 4 metastatic Median age: 11.5 years (RT only group) Median age = 10.8 years (CRT group) 13 males, 3 females	**Surgery:** 16 biopsies **No chemotherapy**	**Local/metastatic disease:** CSI 24–35 Gy (median dose 25 Gy) + tumour boost 15–30.6 Gy (median dose 15.5 Gy)	Median FU = 7 years 10 years EFS = 87.5% OS = 100%	Adjuvant chemotherapy permits RT dose reduction and elimination of CSI (in localised disease) with no significant differences in OS and EFS
	2–3 cycles every 4 weeks: Cisplatin 20 mg/m^2^/day (days 1–5)+ Etoposide 120 mg/m^2^/day (days 1–5)± Bleomycin 15 mg/m^2^ (days 1, 8, 15) **OR** Carboplatin + Etoposide + Ifosfamide **OR** Cisplatin + Etoposide + Cyclophosphamide	**Local disease:** 25–36 Gy (median dose 35 Gy) at primary tumour site **Metastatic disease:** 25–35 Gy at primary tumour site + 20–25 Gy spinal RT (median dose 25 Gy)	Median FU = 3.4 years EFS = 75% OS = 87%	
**Huh et al. 1996 [168]** Retrospective study 32 germinomas: 4 pineal, 14 suprasellar, 2 bifocal, 12 basal ganglia and thalamus, including 3 disseminated cases Median age = 14 years 24 males, 8 females	**Surgery:** 1 total resection, 4 subtotal resections, 11 partial resection, 16 biopsies **No chemotherapy**	CSI/WBRT ± boost RT tumour bed 54 Gy Whole-brain 36 Gy Spinal axis 24 Gy	Median FU = 5.1 years 10-year OS 97% 1 death (persistent tumour) No intracranial or spinal recurrence	Radiotherapy alone yielded favourable results

Abbreviations: CSI = craniospinal irradiation, RT = radiotherapy WVRT = whole-ventricle radiotherapy, WBRT = whole-brain radiotherapy, CR = complete remission, FU = follow-up, OS = overall survival, CRT = chemoradiotherapy, SIB = simultaneous integrated boost, COG = Children’s Oncology Group, EFS = event free survival, FR = focal radiotherapy, PFS = progression free survival, RFS = relapse free survival, HDC = high dose chemotherapy, DFS = disease free survival, ICGCT = intracranial germ cell tumour, NGGCT = non-germinomatous germ cell tumour, 3D PT/3D CRT = three-dimensional conformal proton radiation, IMRT = intensity-modulated photon radiotherapy, SIOP = Société Internationale d’Oncologie Pédiatrique, β-HCG = β -human chorionic gonadotropin, STGC = syncytiotrophoblastic giant cells, CDC = conventional dose chemotherapy, ICE = ifosfamide + cisplatin + etoposide, PBSCT = peripheral blood stem cell transplantation, PEB = cisplatin, etoposide, bleomycin, EP = etoposide, cisplatin, CARE = carboplatin, etoposide, IMPT = intensity-modulated proton therapy with pencil beam scanning, PR = partial remission, ACSR = autologous stem-cell rescue, PD = progressive disease, CNS = central nervous system, GCT = germ cell tumour, * = specification.
